# The General and Special Education of Dentists

**Published:** 1882-02

**Authors:** 


### THE GENERAL AND SPECIAL EDUCATION OF DENTISTS.

In our leading editorial in the November number of the *Independent Practitioner* we stated the views held by us on the present standing of the question of medical education. Regarding dentistry merely as a branch or specialty of general medicine, we hold that in certain of the general branches the education of medical and dental students should be similar. At a certain point of the curriculum the training of the two classes of students should diverge and become special. When this divergence of studies should take place we shall try to indicate in this paper.

As regards the preliminary examination the requirements should be the same for the two classes of students. The dentist must be able to understand the laws of physics and chemistry as well as the medical student, and he should be on the same plane with him as regards general culture.

A knowledge of chemistry, anatomy and physiology is as important to the dentist as to the physician. The first and third should be taught throughout in a similar manner to both medical and dental students, the lectures and laboratory work being the same for both. In anatomy the lectures should also be the same, while the practical work in the dissecting room should here be specialized, the dentist devoting most of his time in the dissecting room to the study of the parts with which he will in his future practice be more immediately concerned.

General pathology should not be distinctively medical or dental, for the processes of inflammation hypertrophy, atrophy, degeneration and new-formation do not essentially differ, whether they occur in the mouth or in the genital organs Special pathology should, however, be specialized as much as possible, for the dental student would profit little by attending the lectures on ophthalmology, gynæcology, or any of the other specialties except dentistry. He must learn all about the diseases affecting the mouth, teeth and jaws, and be able to treat these diseases and obviate their unpleasant results. It matters little whether he knows anything of internal urethrotomy or hystero-trachelorraphy, or whether he is entirely ignorant of these advances in modern special surgery.

Then, instead of the medical student's special training in surgical operations or obstetric work, the dental student must, by demonstration and practice, become practically acquainted with the operative and artificial branches of dentistry. His instructions in these should begin from the time he enters the dental infirmary, for they are to him what the hospital work and operations are to his brother of the medical school.

The question now remains how best to secure the most thorough acquirement of this knowledge. It is our belief that a similar plan to that urged in relation to the education of medical students should be pursued in dental schools. The general branches, anatomy, physiology, chemistry, materia medica, with practical exercises in oral surgery, laboratory and dissecting room should be taught the first year, and the amount of knowledge acquired tested by an examination at the end of the term. The second year's course should be principally occupied with special pathology and operations. Both the theory and practice of operative and artificial dentistry should be taught throughout both years, and as much time as possible be given to general practice.

Habits of cleanliness—of body, clothing, and surroundings—should, of course, have been formed before the student enters the college, but those who, like the writer, have had experience as an instructor in a dental school, know how often this important part of the preliminary education of a dentist is neglected. It is therefore incumbent upon the teacher to constantly enforce upon the student, both by precept and example, the necessity of being scrupulously clean in all things. He should be impressed with the fact that thorough cleanliness is essential to success in dentistry above all professions.

If the methods of medical and dental teaching, which we have attempted to outline, were adopted, it is believed that it would considerably raise the standard of medical education in this country. At the same time it would involve no radical or injurious change to the colleges; on the other hand, it would necessitate harder study on the part of the students, and better teaching on the part of the teachers.---*Independent Practitioner*.

### CORRESPONDENCE.

JANUARY 23, 1882.

DR. TAFF,

*Dear Sir* :—

Will you please inform me when and by whom the first false teeth were made, and in what year first used?

Yours Respectfully,

T. M.

Whether the question has reference to the first production and use of artificial teeth in this country only, does not appear in its construction, but I will here give the evidence upon the subject so far as it is apparent in the literature of the profession.

Delabarre states that an apothecary of St. Germani, named Duchateau was the first to make porcelain teeth. This discovery, he says, was communicated to the Royal Academy of Surgery in 1776. This statement would seem to require corroborating evidence in view of the reports hereto appended.

The statement of Delabarre seems in history to have little or no support further than possibly a mere suggestion. Nothing of a practical nature appeared from this source. The first practical results in the manufacture and use of porcelain teeth were attained between the years 1785 and 1789, by M. Dubois De Chemant, of Paris, France. At, or even after the latter period, M. De Chemant went to London, and spent at least a part of his time there making and introducing his mineral teeth.

In order to give a correct idea of what M. De Chemant did, and the views taken of it by committees appointed to examine and investigate, and report upon the alleged discovery, two such reports are given below, the one made to the Royal Academy of

Sciences, and the other made by commissioners, appointed by the Faculty of Physic, of Paris. And also an extract from the Register of the Faculty of Medicine in the University of Paris:

*Report of the* Academy of Sciences *concerning the Teeth, and sets of Teeth, of the new composition of* M. Dubois De Chemant. *Extracted from the Registers of the Royal Academy of Sciences, the* 10*th June*, 1789.

"M. DARCET and I have been charged to examine the teeth, and sets, of a new composition, which M. Dubois De Chemant has presented to the Academy, and to give in an account of them. The company has been able to judge as we have, that those teeth and sets very nearly imitate nature, as well by their form and colour as by the portions of artificial gums which support them, and to which M. Dubois De Chemant also gives a very great likeness to natural gums. But what merits for them a considerable preference beyond all those which have been composed hitherto, is, that they are of a hard substance, upon which the saliva and the particles of food which remain in the mouth, have no *effect*; whereas the others, made of animal substances, and little resembling natural teeth, are easily spoiled, acquire a dirty colour, and contract a smell as *offensive* as *prejudicial* to the health. The matter which M. De Chemant makes use of is a mineral paste, to which, after many essays, he has found means of giving a colour like to that of the teeth which he means to supply. He can mould it into any form so as to make whole sets, half sets, either for the upper or lower jaw; portions of sets, when there remain above or below teeth, which may be preserved, single, double, treble, or quadruple teeth, as necessity requires. The whole sets are put in motion by means of springs, of M. De Chemant's invention, which are very different from those used heretofore, and which not only separate the parts when the jaws are distended, but also allow the side motions. These springs are applied to both sets, even to the upper, in a manner as simple as it is ingenious. A mechanism equally simple joins the parts of sets to the natural teeth which remain; and single, double, or treble teeth fit with the greatest facility, because M. De Chemant has found means of boring his paste so as to place pins in them, and to make any slides he pleases.

"His manner of taking measure of the teeth which he intends to replace, adds greatly to the merit of his invention. His process is such, that each piece is moulded, as it were, for the place which it is to fill; and as for the whole sets, half sets, or any other portion whatsoever, their base receives and surrounds the edges of the gums, or the part on which they are applied, so as to render their position very solid, and to prevent the painful pressure they may otherwise occasion. By this process he can preserve, as long as he pleases, the moulds of all his pieces, and can take very exact and precise measures of persons at a distance whom he never saw; and provided he be informed exactly of the colour of the remaining teeth, he is sure to send pieces which will fit with the greatest exactness, as well as if he had taken the measures and placed the teeth himself.

"M. De Chemant's paste is very solid; it cannot be broken between the hands, without employing very great strength. The substance of it produces fire with steel; it is not affected by acids. The weight of it is less than that of porcelain. M. Briffon, who has been pleased to determine it, found that a cubical inch of it weighed one ounce, two gros*, and sixty-nine pennyweights; whereas the lightest china of Seve, of the seventeen kinds which he tried, weighs one ounce, three gros, and nine grains.

Having examined the teeth and sets of teeth made by M. De Chemant, after seeing the manner in which he takes his measures and forms his moulds, having inquired into the springs and the means he employs to adapt his pieces, in order to justify the confidence laid in us by the Academy, we thought proper to see some pieces placed on; we therefore went ourselves to the houses of different persons who make use of them, and who have consented to be visited and to answer our questions. We have seen teeth of every kind. The persons to whom M. De Chemant conveyed us are all of a distinguished rank, and of course beyond all suspicion of any other views in what they told us than those of doing justice to truth. They assured us they felt no sort of inconvenience from the pieces they make use of, and that they became accustomed to them in a very short time, and with ease. They use them to eat, and find them of assistance in the action of chewing as well as of speaking, at the same time that they remove the deformity arising from the want of teeth. We have seen no person whose pieces have either lost their colour or received any other hurt, by any bits falling off; and though that should happen, and some scraps should mix with the food, we think we may affirm, that nothing dangerous could result from it, and that those particles may be swallowed without any more danger than particles of bones of fish or any other hard substance which we are liable to swallow in eating. There is then nothing to apprehend from the teeth or sets of teeth made by M. De Chemant, which moreover possesses all the advantages that can be desired.

"The Academy will, no doubt, permit us to conclude, from what has been said, that the artificial teeth and sets of teeth, of M. De Chemant, deserve being approved by it, and that it would be proper that history should mention the happy application he has made of a hard and incorruptible matter to an end so useful as that of supplying the want of lost teeth.

(Signed)

"D'ARCET and SABATIER.

* A gros is the eighth part of an ounce.

"*At the Royal Academy of Sciences, June* 10, 1789."

"I certify the present extract is agreeable to the original, and to the judgement of the Academy. (Signed)

"*Paris, June* 21, 1789." "The Marquis De CONDORCET.

*Report of the* Commissioners, *appointed by the* Faculty *of* Physic *of* Paris, *to examine the new Teeth and Sets of Teeth, invented by* M. De Chemant.

"MR. DEAN,

"We have examined the new artificial teeth and sets of teeth, which the Sieur De Chemant forms of a paste of his composition, which he hardens by the fire; their hardness is so great that they long resist the hammer, and often produce fire, like flint struck with steel: no kind of acid can dissolve them; a piece, representing the whole set of the upper jaw, may be thrown against the floor, without breaking.

"The sets of the upper jaws are of one entire piece; the teeth are not separated by real interstices; they are represented each according to its natural form, and a coloured shade seems to separate them. The gums are also perfectly imitated: on the edges of these sets, are some inequalities which represent the upper extremities of the different kinds of teeth.

"By the form which the Sieur De Chemant gives his teeth, they perfectly resemble nature; he has also discovered the means of giving them the colour of the natural teeth, to which they are substituted, so that they cannot be distinguished from the natural teeth of the person who wears them; and as the substance of which they are made is incorruptible, it loses none of its properties by time.

"The whole set, composed of an upper and lower jaw, is jointed by a spring, invented also by the Sieur De Chemant, by means of which both jaws move with great facility, and without any troublesome or inconvenient resistance to the bearer, in their different movements. We have seen a person wear an upper set, which fitted perfectly, was no ways incommodious to the patient, who, whenever he spoke or laughed, seemed to have a beautiful set of teeth. We have also seen several teeth joined together, in the mouth of a person, who maybe relied on, and who assured us, he could eat with those artificial teeth, as well as he formerly used to do with his natural ones.

"This invention of M. De Chemant's seems to us, to unite all the advantages, which persons who want artificial teeth can wish for. When he is about to supply the defect of one or many contiguous teeth he takes with his paste, the length of the space to be filled, and the form of the edges of the gums, with the greatest precision; he then forms a piece which fits so exactly as never to incommode the wearer. The hardness of the composition is such, that it never wastes by mastication, and its incorruptibility prevents it from being dissolved either by solid or liquid food: as the teeth are not separated in their length, no parts of the aliments can remain amongst them.

"Hitherto dentists had no other means of supplying the want of teeth, than the bony substances of different animals, of which they formed either single teeth, or many teeth together or whole sets of teeth; they took a part of a bone to form the piece they wanted, and made use either of a file or a saw to work it: when they intended to make a set for either, or for both jaws, they gave a piece of bone the proper shape, and then marked with a saw on the surface, a line to imitate the space which commonly divides the teeth from each other; these teeth, particularly those of the fore part of the mouth, rather resembles the keys of a spinnet than real teeth, and had a considerable opening, as well at their upper end as in their whole length; parcels of food remained in them, fermented in the mouth, corrupted and exhaled an infectious smell, as noxious to the patients themselves, as intolerable to those, to whom they spoke too closely.

"We think it proper to observe, that the file and saw employed to shape those teeth or sets of teeth, made of bone, by opening a great number of pores in them, where the juices of the mouth and aliments could penetrate, disposed them to corrupt in the mouth: it is a fact, that those bones softened, corrupted, and wore away in the mouth. We have seen on the same set, two exfoliated teeth, and we lay before the faculty, an old set, which we received from the Sieur De Chemant, which became soft and black in the mouth of the person who wore it.

"M. De Chemant's teeth have none of the inconveniences of those made of bone: they have the advantage of resembling perfectly the form of every kind of teeth, of representing the intervals without leaving any void space, of representing the gums, and fitting so exactly on the edges as never to be troublesome to the wearer. We therefore think that the faculty should admit the discovery of M. De Chemant, as an invention which does much honour to its author, and must be very useful to those who are in need of the assistance of this new art

(Signed)

"Descemet, Baget, and Petit-Radel."

*Extracted from the Registers of the Faculty of Medicine in the University of Paris.*

"In the year one thousand, seven hundred and eighty-nine, on Monday the second day of March, the Faculty of Medicine assembled at five o'clock in the afternoon, in its upper schools, after having heard the report made to them by M. M. Descemet, Baget, and Petit-Radel, whom they had charged to examine the artificial teeth and sets of teeth, proposed by M. De Chemant, Surgeon and Dentist, has been unanimously of opinion, agreeably to the said report, to approve the same artificial teeth and sets of teeth, composed of a paste which the Sieur De Chemant hardens by fire, so that those pieces unite, at the same time, beauty, solidity, convenience, and salubrity, qualities acknowledged by the Commissioners, as well by the trials made upon specimens presented by the inventor, as by what they observed with persons who have made use of them, and I have concluded in approving the sentiments of the faculty.

"EDME. CLAUDE BOURU, *Dean.*

"On the part of M. M. the Deans and Doctors Regent of the Faculty of Medicine of Paris, I have affixed the small seal, the 5th of March, 1789.

(Signed)

CRUCHOT,

*First Apparitor and Register Keeper of the said Faculty, in the University of Paris.*

Of his success in the application of his porcelain dentures, M. De Chemant says, in 1796:

"If the names of more than three thousand persons could with propriety be mentioned, who make use of the new invented teeth, which are the subject of this Dissertation, this approbation would fully prove the superior advantages of teeth formed of mineral paste; but preserving inviolate those bounds the confidential duties of the profession prescribe, no patient's name can be pronounced without the most express permission."

In order that he might the more fully establish his priority of invention and discovery, M. De Chemant took out letters patent in both France and England, which would certainly have been resisted had any other had a prior claim. And in addition to this there is hardly a shadow of probability that the Committees whose reports are here given, as well as several others that might be given, were imposed upon, or ignorant of the past, if so important discoveries had been before made.

The first porcelain or mineral teeth in the United States were brought from France about 1817, by Dr. A. A. Plantou, and used by him in his practice in Philadelphia. These teeth were said to have been very inferior in appearance and consistence, were probably much inferior to those made by M. De Chemant. Dr. C. W. Peale, of Philadelphia, and Dr. H. Villers, of Boston, about this period, engaged in the manufacture of porcelain teeth.

MANSFIELD, O., Jan. 23, 1882.

EDITOR DENTAL REGISTER:

Dear Sir:—I regard the following as one of the very extreme cases of replanting. If you think it will be of interest to your readers please publish.

On Thursday night, Jan. 4th, Jno. Kendall, aged about seventeen, and residing six miles north of here, was blowing a horn, a kick was administered on the large end of it, driving it into his mouth with such force as to knock out the superior left central and lateral incisors, carrying away the anterior wall of the alveolus and cutting entirely through the lip. The teeth laid in the snow several hours, when some of the family went back with lanterns and found them. Next day, about 10 o'clock, they brought the young man to me, his mouth badly swelled, having the teeth in a paper, they having been out of the mouth fifteen hours. Having done some replanting, the friends demanded that these teeth be put back. I had very little hope of a successful result, but, under the circumstances, concluded to do the best I could. On examination I found the outer wall of the sockets entirely gone, the gum had collapsed and filled them. After scraping the roots of the teeth and filling the fangs from the apex with gold. Remembering the Thomsonian adage, "Heat is in life, and cold is death," I put them in tepid water until they go thoroughly warmed. While they were in the water I removed a couple of loose pieces of bone, cut and pressed out the gum, cleaned out the socket, first with warm water and then with cold, then pressed the teeth firmly against the remaining wall, tied them carefully so they could not move; in tying them they dropped a little, I took a flat file and pressed them back and held them for half an hour. Prescribed abstemious diet and quiet. Saw my patient every day, when I painted the gums with tincture of Iodine, and at the end of four days allowed him to go home. Saw him no more until the 20th, the teeth had seemingly healed by first intuition; swelling gone, and the teeth firm, and the operation a success.

Fraternally Yours,

T. G. BRISTOR

